# Stronger Together. Poly(Styrene) Gels Reinforced by Soft Gellan Gum

**DOI:** 10.3390/gels8100607

**Published:** 2022-09-22

**Authors:** Dariya Getya, Ivan Gitsov

**Affiliations:** 1 Department of Chemistry, State University of New York—ESF, Syracuse, NY 13210, USA; 2 The Michael M. Szwarc Polymer Research Institute, Syracuse, NY 13210, USA; 3 The BioInspired Institute, Syracuse University, Syracuse, NY 13244, USA

**Keywords:** gels, Gellan Gum, semi-interpenetrating networks, amphiphilic, conetworks

## Abstract

This study targets the synthesis of novel semi-interpenetrating networks and amphiphilic conetworks, where hydrophilic soft matter (Gellan Gum, GG) was combined with hydrophobic rigid poly(styrene), PSt. To achieve that, GG was chemically modified with 4-vinyl benzyl chloride to form a reactive macromonomer with multiple double bonds. These double bonds were used in a copolymerization with styrene to initially form semi-interpenetrating networks (SIPNs) where linear PSt was intertwined within the GG-PSt conetwork. The interpenetrating linear PSt and unreacted styrene were extracted over 3 consecutive days with yields 18–24%. After the extraction, the resulting conetworks (yields 76–82%) were able to swell both in organic and aqueous media. Thermo-mechanical tests (thermal gravimetric analysis, differential scanning calorimetry, and dynamic mechanical analysis) and rheology indicated that both SIPNs and conteworks had, in most cases, improved thermal and mechanical properties compared to pure poly(styrene) and pure GG gels. This crosslinking strategy proved that the reactive combination of a synthetic polymer and a bio-derived constituent would result in the formation of more sustainable materials with improved thermo-mechanical properties. The binding ability of the amphiphilic conetworks towards several organic dyes was high, showing that they could be used as potential materials in environmental clean-up.

## 1. Introduction

Poly(styrene) (PSt) is one of the most widespread petroleum-based polymers to be used in various applications [1]. Materials made of PSt not only have a low cost, but also possess a good balance of toughness, rigidity, and transparency [2]. The shift of usage towards “greener” and renewable materials has also led to an increased interest in sustainable, eco-efficient, and annually renewable resources [3]. Such sustainable and “green” polymers are produced partially or entirely from natural renewable feedstocks. For example, starch, cellulose, alginic acid, and chitin have found increased use as fillers, compatibilizers, and additives [4,5,6,7]. The combination of a synthetic polymer and renewable constituent led to the development of the next generation of materials where the naturally derived components are inserted in the mix, producing new polymeric materials with new properties [8].

Multiple natural polymers are being utilized to improve and adjust the physical properties of synthetic polymers. Starch (a polysaccharide) is one of the most widely used for this purpose. Pimentel and co-workers created a blend of PSt with cassava starch using a natural plasticizer—Buriti oil [9]. This polymer blend, however, underwent a phase separation, and had worse thermal stability compared to pure PSt. Berruezo et al. tested another approach and melt-blended starch and PSt with similar results [10]. Ismail et al. synthesized a superabsorbent composite material composed of waste PSt, starch, and acrylic acid that was prepared using emulsion polymerization [11]. They showed that adding bentonite to the polymer network leads to an increase in thermal stability.

Cellulose is among the most extensively used naturally derived additives as a reinforcing phase in nanocomposites [12,13]. It is known that addition of cellulose nanocrystals (CNCs) or nanofibers (CNFs) to a polymer matrix improves the mechanical and thermal properties of the resulting composites [14,15,16] or networks [17,18]. However, due to its hydrophilicity, raw cellulose does not disperse evenly in the blend [19], thus limiting the application of such materials. In our most recent paper, we reported a new approach to overcome this limitation through formation of semi-interpenetrating networks (s-IPNs) where modified cellulose fibers are covalently fused into a PSt network [20]. This method minimized, to a large extent, the agglomeration of the cellulose fragments in the matrix. The networks also showed good swelling properties in chloroform and exhibited improved thermal and mechanical properties [20].

**Figure 1:**
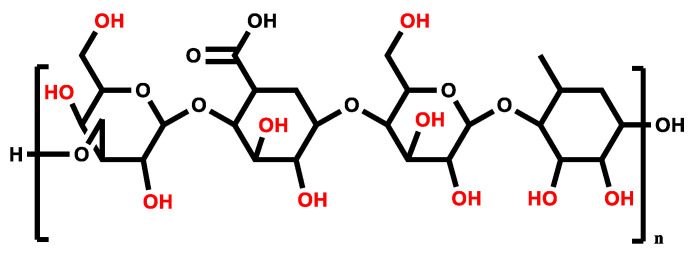
Chemical structure of the Gellan Gum repeating unit.

Gellan Gum (GG) is a polysaccharide that is broadly utilized in the food industry [21]. Back in 1988 it was accepted in Japan as a ‘natural food additive’, and since then, it has had a history of safe use in the food industry, as noted by numerous food regulatory agencies, including the Food and Drug Administration [22]. Recently, GG has been gaining more attention as a material for tissue and cartilage engineering [23,24,25,26], as a potential wound dressing material [27], and even as a chromatographic medium for purification of biomolecules [28]. Figure 1 shows the chemical structure of low acyl GG. The main chain consists of four repeating carbohydrates that include two d-glucose carbohydrates, one l-rhamnose, and one d-glucuronic acid moiety. Due to its helical structure and the presence of carboxylic groups, GG can form hydrogels upon addition of mono- or divalent cations, but they are somewhat brittle and have poor mechanical strength, poor stability under physiological conditions, and a small temperature range to self-assemble [29]. Improvement strategies have been proposed, where GG is combined with different natural or synthetic polymers such as agar, chitosan, poly(vinyl chloride), and poly(methyl methacrylate). For example, Karthika and Vishalakshi have synthesized GG-grafted-poly(2-dimethylamino ethyl methacrylate) by free radical polymerization as an adsorbent for the removal of anionic dyes from dye-contaminated water samples [30]. N,N’-methylenebisacrylamide (MBA) was used as a crosslinker in the three-dimensional network [30].

While GG found reasonable applications in the food and pharmaceutical industries, it has been surprisingly overlooked as an ingredient for polymers’ structure enhancement. To the best of our knowledge, no reports have been published on the synthesis of composite networks, where hydrophobic synthetic polymers such as PSt were used together with GG. Here, we propose the synthesis of a novel gel material where soft matter such as modified GG is incorporated into a network through copolymerization with styrene. The resulting networks are transparent in a swollen state, indicating a homogeneous GG dispersion throughout the PSt matrix. The PSt-GG gels also have improved mechanical and thermal properties, good swelling characteristics in chloroform and water, and notable selective binding ability towards several anionic and cationic dyes.

## 2. Results and Discussion

### 2.1. Synthesis of the Macromonomers—Styrene-Modification of Gellan Gum

**Scheme 1:**
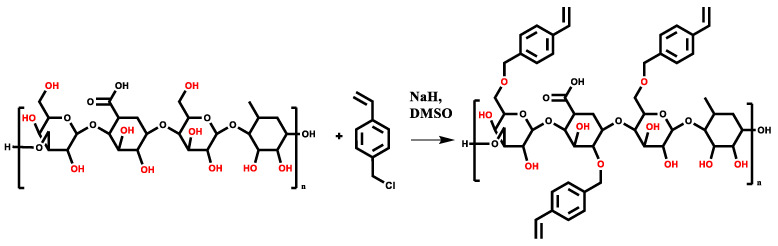
GG Williamson ether reaction with 4-VBC.

The successful incorporation of styrene moieties in a carbohydrate (cellulose) was reported in our previous work [20]. The same approach was used in this work for the GG modification. 4-Vinyl benzyl chloride (4-VBC) reacted with NaH-activated hydroxyl groups present in GG molecule, leading to the formation of a multi-functional macromonomer that now contains multiple double bonds, Scheme 1. These double bonds can now participate in further (co)polymerization reactions.

**Figure 2:**
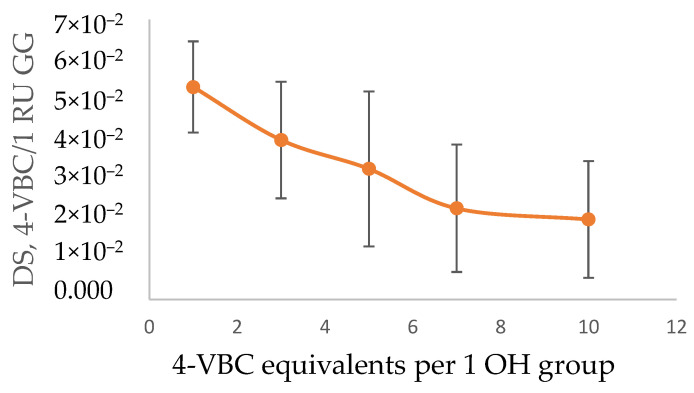
Degree of substitution (DS) of the GG—attached 4-VBC groups per one repeating unit (RU) in GG.

The amount of attached methyl styrene moieties on the GG chain (degree of substitution, DS) can be controlled by varying the 4-VBC amount used in the modification reaction. Five component ratios were tested: 1, 3, 5, 7, and 10, i.e., 1, 3, 5, 7, and 10 4-VBC molecules per one repeating unit, respectively. They are referred to as GG-1, GG-3, GG-5, GG-7, and GG-10. Figure 2 shows the DS for each reagent ratio derived from the calibration curve (Figure S1). In the best-case scenario, the reaction leads to the incorporation of one double bond moiety in every 19th repeating unit (more than 26,000 vinylbenzyl groups per single GG chain). Interestingly, the DS decreases with the increase in 4-VBC amount in the reaction mixture, regardless of the large NaH excess (see ‘Material and Methods’ for details). A possible explanation for this fact could be that 4-VBC has limited accessibility to all OH groups, especially those in the bulk of GG due to the large GG molecular mass (typical value ~500 kDa [29]) and increased steric hindrance after the initial modification on the surface of the GG chains.

**Figure 3:**
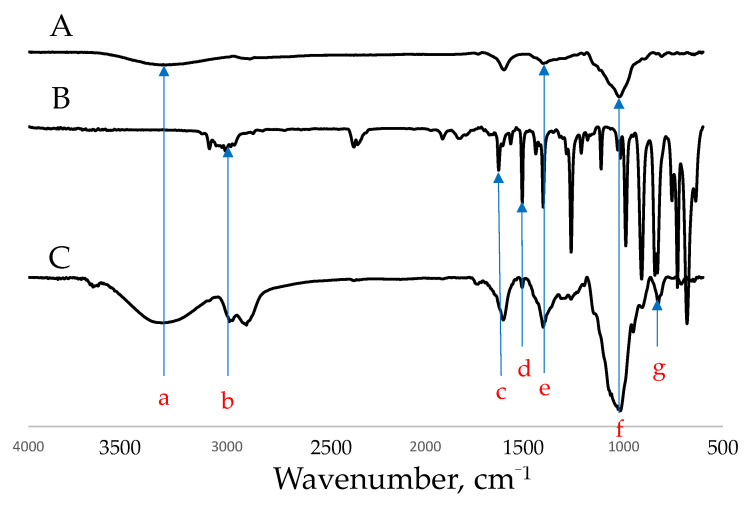
Stacked FT IR spectra of GG (A), 4-VBC (B), and VBzGG-3 (C).

FT IR analysis of modified GG confirmed the attachment of a methyl styrene moiety to the GG chain. Characteristic peaks for carbohydrate and poly(styrene) were assigned and described in the literature [26,31], and they were reliably identified in the GG-m IR spectra (Figure 3). O-H stretching vibration was seen at 3500 cm^−1^ (Figure 3a). Interestingly this band was stronger in GG-m compared to pure GG. Most probably, the introduction of methyl styrene moieties disrupts the hydrogen bonds between multiple OH groups along the GG chains, with the remaining free hydroxyls experiencing signal enhancement in the GG-m. The C-H aromatics stretching vibration from the methyl styrene groups appeared around 3000 cm^−1^ (Figure 3b). C=C aromatic bands could be observed at 1602 and 1540 cm^−1^ (Figure 3c,d). Symmetrical deformations of CH_2_ groups appeared around 1406 cm^−1^ (Figure 3e). The strong signal in GG and GG-m at 1000 cm^−1^ was caused by a combination of C-O-C and primary alcohol CH_2_OH stretching (Figure 3f). Lastly, the di-substituted benzene =C-H out-of-plane vibration band was visible at 840 cm^−1^ (Figure 3g). These bands confirmed the incorporation of vinylbenzyl (VBz) moieties into the GG structure.

**Figure 4:**
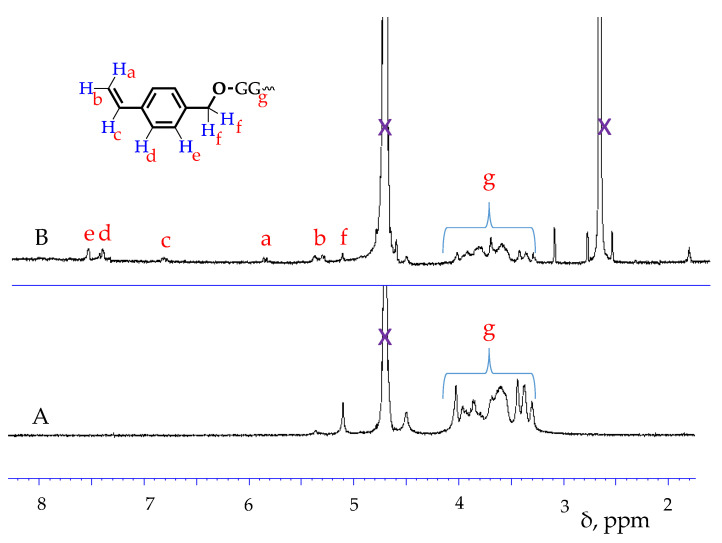
NMR spectra of GG (A) and VBzGG-3 (B) in D_2_O. X—solvent peaks.

**Figure 5:**
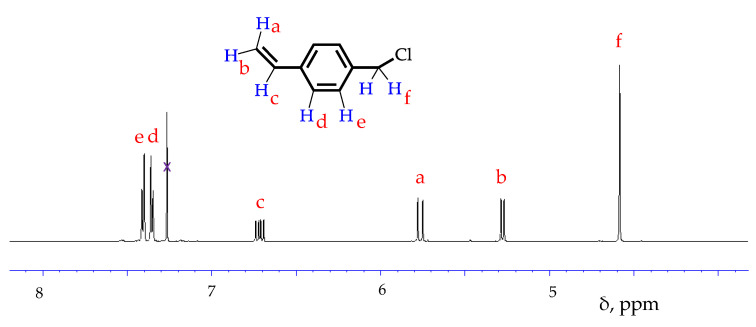
NMR spectrum of 4-VBC in deuterated chloroform. X—solvent peak.

Figure 4 shows the ^1^H NMR spectra of GG and VBzGG-3 in D_2_O. The GG chain signals are in the area between 3.2 and 4.2 ppm [32]. The GG-m spectrum has signals that come from the GG chain, but in addition, contains peaks that are originating from incorporated methyl styrene groups, which is the result of the modification reaction. 4-VBC is not soluble in water, so its ^1^H NMR spectrum cannot be compared directly with GG, but its structure is well studied in various other solvents [33,34] (Figure 5).

### 2.2. Preparation and Characterization of GG-m/PSt Gels

**Scheme 2:**
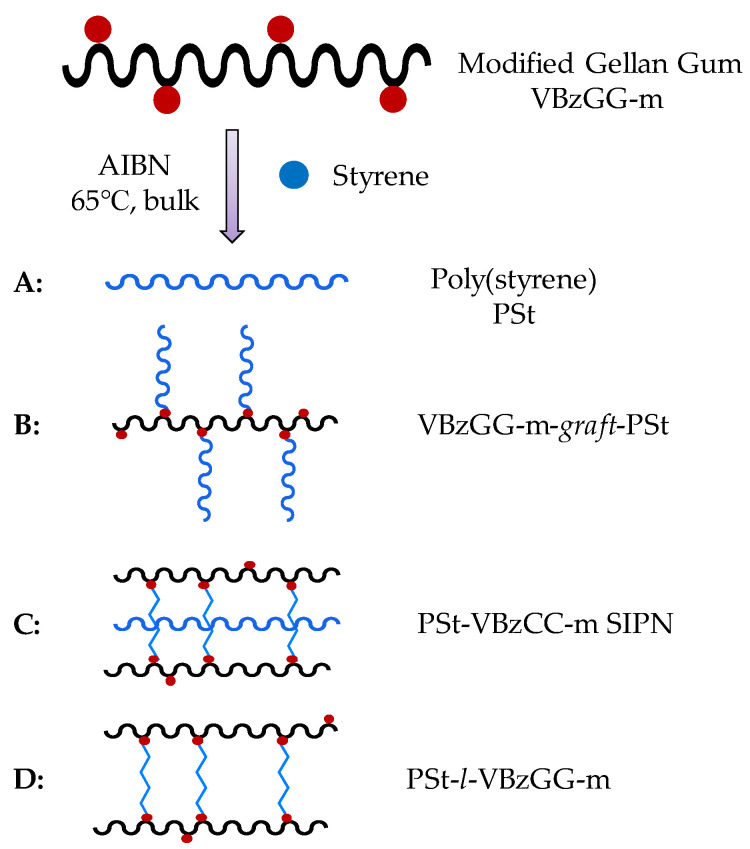
Possible processes occurring during copolymerization of GG-m and styrene at 65 °C. Black line—GG, red dots—methyl styrene groups, blue dots—styrene; light blue wavy lines—PSt homopolymer and/or grafted PSt, zigzag blue lines—crosslinked PSt segments. A—St homopolymerization, B—St grafting copolymerization onto VBxGG-m, C—simultaneous St homopolymerization and St crosslinking copolymerization with VBzGG-m, D—St crosslinking copolymerization with VBzGG-m.

VBzGG-m/St bulk radical copolymerization was initiated by 2,2′-azobis(isobutyronitrile), AIBN (Scheme 2). There are several products that could be formed during this process: PSt homopolymer (Scheme 2A), bottle brush-like copolymer (VBzGG-m-*graft*-PSt, Scheme 2B), semi-interpenetrating network (PSt-VBzGG SIPN, Scheme 2C), and conetwork (PSt-*l*-VBzGG-m, Scheme 2D). The possibility for VBzGG-m homopolymerization is statistically very low due to the small content of the macromonomer in the copolymerization mixture.

**Figure 6:**
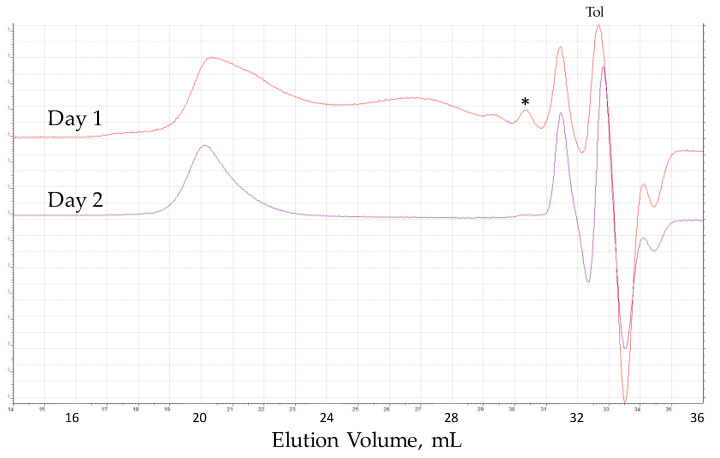
Size-exclusion chromatography dRI traces of products extracted with CHCl_3_ from PSt-VBzGG-3 SIPN over 24 and 48 h. (*)—unreacted styrene, (Tol)—toluene flow rate marker.

**Figure 7:**
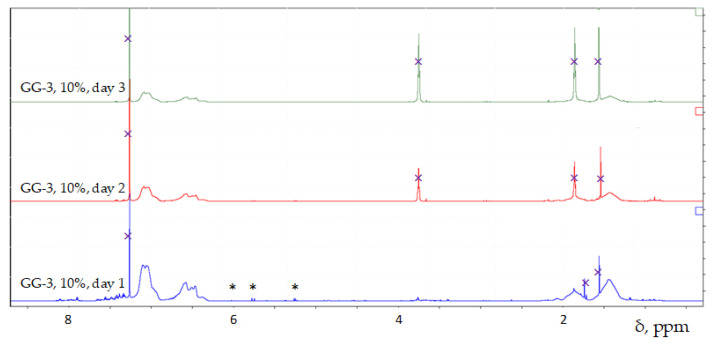
^1^H NMR spectra of products extracted with CHCl_3_ PSt-VBzGG-3 1 wt% SIPN over 24, 48, and 72 h. (GG-3)—PSt-VBzGG-3 1 wt% SIPN. (*)—unreacted styrene, (×)—solvent signals.

The analyses of the products, isolated after the 3-day CHCl_3_ extraction of the solid reaction mixtures, provided information about the outcome of the copolymerization process (Figure 6 and Figure 7). The chloroform-soluble fraction isolated after 24 h contained a mixture of unreacted styrene (marked with ‘*’ in both figures), styrene oligomers, and pure PSt (Figure 6 and Figure 7). Expected extracts from the next two days contained only pure PSt of higher molecular mass, which was able to migrate into the liquid phase through the fully swollen networks (Figure 6 and Figure 7). The extracted PSt from all experiments had broadly monomodal molecular mass distribution (Figure S2), indicating that the St homopolymerization occurred as a uniform propagation process. It should be noted that signals attributable to GG could not be seen in the FT-IR and NMR spectra of the chloroform-soluble fractions (Figure 7). Thus, it is reasonable to assume that PSt grafting onto GG (Scheme 2B) was negligible or not existing. The FT IR spectra of the initial copolymerization mixture and the products remaining after three CHCl_3_ extractions contained all characteristic PSt and GG vibration bands, Figure S3. The chromatographic and spectral analyses provided mutually supported evidence for the initial formation of a semi-interpenetrating network (s-IPN). In this network, styrene formed the inter-crosslink PSt segments between multiple GG-m and also yielded linear PSt homopolymer intertwined between the crosslink junctions that can be extracted (Scheme 2C). After the extraction, the remaining products were not soluble in DMSO (a common solvent for both constituents) and swelled in chloroform and water, giving us sufficient justification to assign them to the class of amphiphilic conetworks (Scheme 2D) [35,36,37,38]. The products after the extraction process will be designated as poly(styrene)-*l*-(vinyl benzyl Gellan Gum) or PSt-*l*-VBzGG-m, where ‘*l*’ denotes “linked by” [39] and ‘m’ signifies the 4-VBC equivalent used in the GG modification.

Three types of SIPN/conetworks were synthesized containing 1, 5, and 10 wt% of VBzGG-m. Table 1 lists the copolymerization conditions, total amount of extractables, conetwork yields, and swelling degree (SD) in chloroform and water. Noticeably, SD (an indicator for the crosslink density) changes depending both on the type and amount of the VBzGG-m macromonomer. Conetworks prepared with VBzGG-1, which had the highest degree of substitution according to UV–Vis analysis, showed the lowest SD. VBzGG-10 has the lowest degree of substitution and the resulting conetworks had the highest SDs. The same trend was observed for the gels prepared using different VBzGG-m amounts. In chloroform, conetworks produced with 1 wt% of VBzGG-m swelled more than ones prepared with 10 wt%. At the same time, the molecular mass of the extracted PSt decreased with the increase of the VBzGG-m amount in the copolymerization mixture (M_w_ = 159,600 Da, 147,200 Da, and 106,500 Da for VBzGG-3 1 wt%, 5 wt%, and 10 wt%, respectively; Figure S4), a clear sign that styrene was preferentially consumed by the crosslinking reaction. As expected, the swelling ability in water is reversed due to the increased weight% of the hydrophilic ~500 kDa GG.

**Figure 8:**
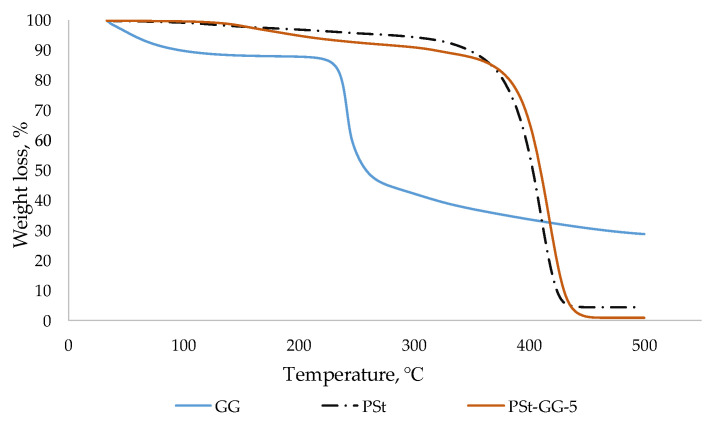
Thermograms of GG (blue line), linear PSt (dotted line), and PSt-VBzGG-5 SIPN (orange line).

The thermal decomposition graphs obtained by the thermal gravimetric analysis (TGA) for the GG, extracted PSt, and PSt-VBzGG-5 SIPN are shown in Figure 8. For clarity, only one PSt-GG-5 SIPN curve (at 1 wt%) is shown, as all of them overlap on the graph. The other TGA curves are included in the Supporting Information, Figure S5. The analysis demonstrated that compared to pure PSt and GG, PSt-VBzGG-m SIPNs had slightly improved thermal characteristics, Figure 8 and Figure S5. The careful examination of the TGA traces revealed that the SIPNs decomposed in two stages. The first stage at 123 °C marks the decomposition onset of GG segments and the second one at 395.4 °C could be attributed to PSt depolymerization onset. Due to the presence of multiple crosslinks, the PSt-VBzGG-m SIPNs decomposed over a slightly higher temperature range (343–460 °C vs. 321–453 °C for linear PSt), despite the presence of the thermally less stable GG.

The glass transition temperature (T_g_) of synthesized networks and heat capacity jump (ΔC_p_) are listed in Table 2 (see also Figure S6). Linear PSt and a PSt/GG physical mixture (St polymerized in the presence of unmodified GG) prepared at identical conditions were used as reference (Table 2, bottom section). The networks were tested before and after the extraction of non-crosslinked linear PSt. The data show that the T_g_ values for several of the conetworks are significantly higher than the T_g_ for linear PSt, while the ΔC_p_ values do not show a well-expressed trend. The increase in T_g_, together with the decrease in the heat capacity jump in the networks, strongly suggest that most of the polymer segments (PSt and VBzGG-m) are immobilized by the crosslinks formed [40,41]. Interestingly, T_g_ for all PSt-*l*-VBzGG-m gels is ~106 °C, well above all s-IPN and the extracted linear PSt, indicating the plasticizing effect of the linear PSt intertwined in the s-IPNs.

The viscoelastic properties of the SIPNs formed (storage modulus, E’ and loss modulus, E”) were analyzed by dynamic mechanical analysis (DMA). They provide information about the elastic behavior (E’) and represent the viscous component (E”) of the materials. DMA also yields data for Tan δ, which is the damping properties indicator.

**Figure 9:**
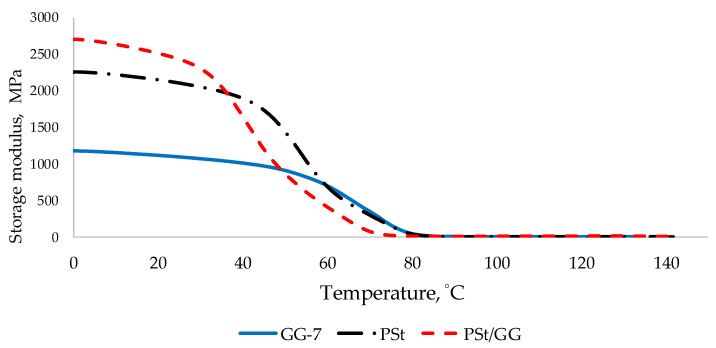
E’ of PSt-VBzGG-7 SIPN (GG-7, solid blue trace), PSt (black trace) and PSt/GG mixture (red dotted trace).

**Figure 10:**
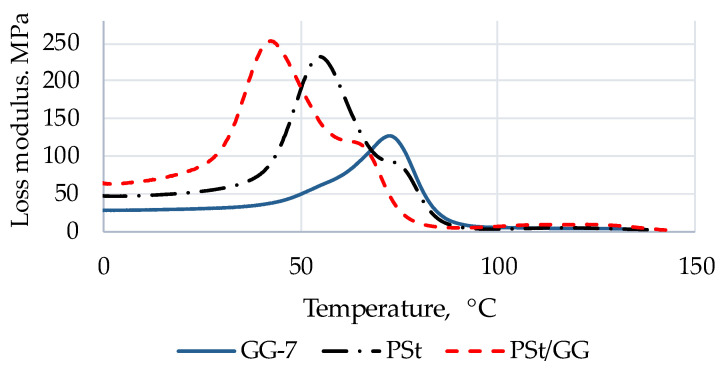
E” of PSt-VBzGG-7 SIPN (GG-7, solid blue trace), PSt (black trace), and PSt/GG mixture (red dotted trace).

**Figure 11:**
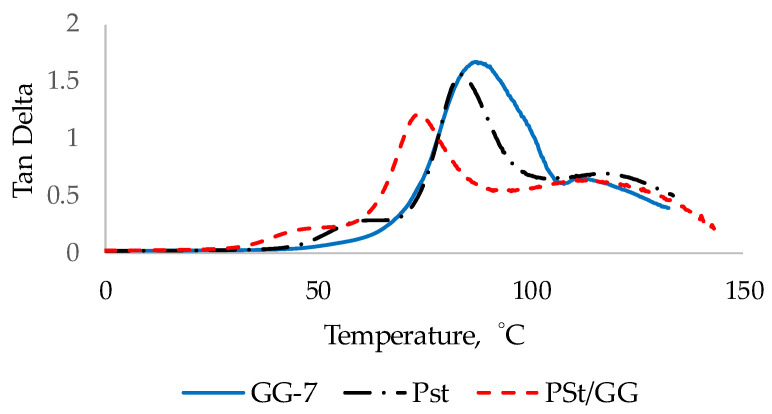
Tan δ of PSt-VBzGG-7 SIPN (GG-7, solid blue trace), PSt (black trace), and PSt/GG mixture (red dotted trace).

Figure 9, Figure 10 and Figure 11 show E’, E”, and Tan δ, respectively, of SIPNs prepared with 1 wt% of VBzGG-7. The analyses with different wt% are included in the Supporting Information, Figures S7–S9. A physical mixture of GG and PSt (St polymerized in the presence of GG) was prepared as a reference material, where GG is not modified and therefore is not crosslinked. SIPNs show improved mechanical properties compared to both linear PSt produced at the same conditions as the networks (initiator amount, time, and temperature) and PSt/GG physical mixture. It was seen that the networks have both storage modulus curves and loss modulus peaks shifted towards higher temperatures. It is noteworthy that the materials produced in this study retain their viscoelastic properties over an extended temperature window compared to pure GG gels swollen in aqueous media [23].

Tan δ is the ratio of E” vs. E’ (Tan δ = E”/E’) and is the damping behavior factor. The samples Tan δ peak shifted to the higher temperatures and the peak itself was wider, meaning that damping occurs at a broader and higher temperature range.

**Figure 12:**
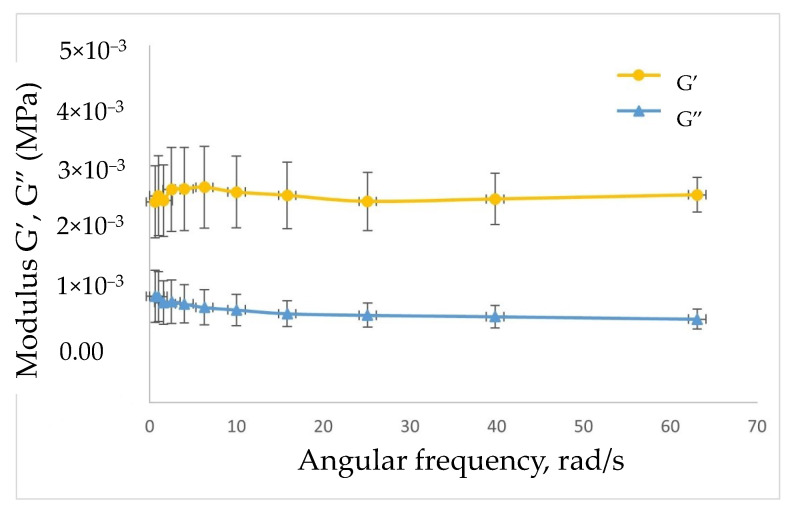
Effect of frequency sweep on the elastic (G’) and viscous (G”) moduli of water-swollen PSt-*l*-GG-5 10 wt%. See ‘Materials and Methods’ for test conditions.

Rheology analysis could provide relevant information on the structural integrity of the conetworks after the chloroform removal of linear PSt. A test of PSt-*l*-VBzGG-7 10 wt% is shown in Figure 12. The observed elastic response to shear deformation is a clear sign for a typical strong gel-like behavior [42] where G’ and G” remained almost parallel over the entire frequency range with the elastic (storage) modulus, with G’ being notably higher than the viscous (loss) modulus G”. The phenomenon of both moduli remaining almost constant over a broad frequency range is caused by the presence of multiple crosslink segments that are able to store the deformation energy over a long timescale [42].

**Figure 13:**
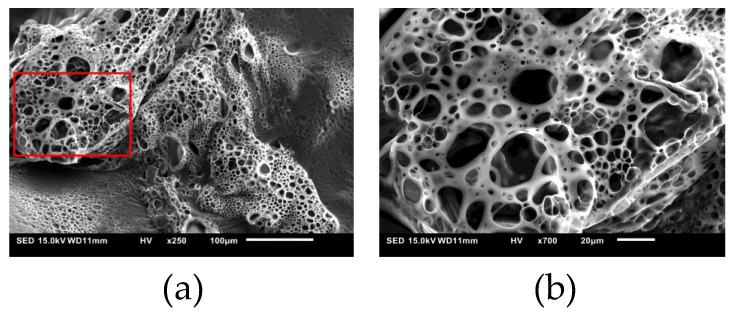
SEM of a PSt-*l*-VBzGG-3 1 wt% gel swollen in CHCl_3_. (**a**) Sample at 250× magnification; (**b**) red-framed area in image (**a**) observed at 1100× magnification. See ‘Materials and Methods’ for analysis conditions.

Surface morphology was studied by scanning electron microscopy (SEM), Figure 13. The SEM images of chloroform-swollen conetworks revealed pores of multiple sizes in the gels, which are rather different from those in PSt-*l*-cellu-mer networks prepared by the same strategy [20]. Despite their irregular appearance and broad size distribution, they more closely resemble the pore structure of hydrogels containing hyperbranched poly(4-VBC), the same chemical moiety incorporated along the VBzGG chain [43]. The morphology of conetworks swollen in water (Figure S10) differed drastically from that of pure GG gels swollen in aqueous media [44]. This is probably caused by the large amount of hydrophobic PSt interlink segments that prevent extensive swelling (see also Table 1).

**Figure 14:**
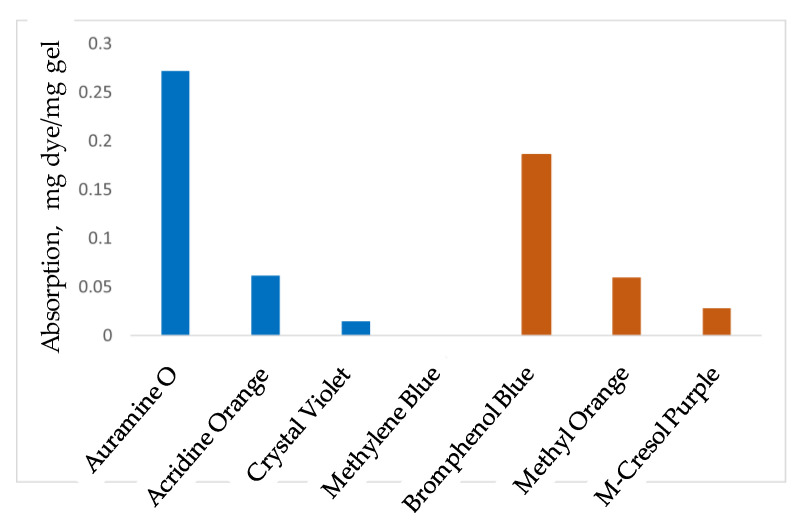
Binding of cationic (blue bars) and anionic (orange bars) dyes in a PSt-*l*-VBzGG-7 10 wt% gel. See ‘Materials and Methods’ for test conditions.

The continuous network of pores is an essential prerequisite for a good absorbent. The binding of several anionic (bromphenol blue, BPB, and m-cresol purple, CP) and cationic dyes (acridine orange, AcOr; auramine O, AO; crystal violet, CV, and methylene blue, MB) was used as an evaluation tool for the absorption abilities of the newly synthesized conetworks. It has been previously reported that GG-based gels can be used as adsorbents for both anionic and cationic dyes from aqueous solutions [30,45,46]. The results from this study are compiled in Figure 14. It is seen that the gels are capable of absorbing both anionic and cationic dyes. The most substantial binding was achieved with the cationic AO and the anionic BPB. Comparing the structures of AO and BPB (Figure S11), gel binding capacity presumably depends not necessary on the negative or positive charges in the dye molecules, but more significantly on their size and their specific interactions, both with the PSt and GG segments in the network matrix.

## 3. Conclusions

The results show that SIPNs and PSt-*l*-GG-m conetworks with improved thermal and mechanical properties can be successfully formed by copolymerization of St and GG modified with 4-VBC. The test data indicate that the SIPNs have significantly improved mechanical and thermal properties compared to pure PSt, even after incorporation of up to 10 wt% of such soft and brittle material as GG. Rheology tests provide proof of how the PSt interlink segments can absorb the deformation energy and thus strengthen the PSt-*l*-GG-m conetworks. Surface morphology studies reveal their extended porous structure after 24 h swelling. The ability of these gels to expand in both organic and water media, combined with their porosity, opens a wide range of potential applications. The selective adsorption of different anionic and cationic dyes exemplifies the potential of the gels to serve as reinforced materials for environmental clean-up.

## 4. Materials and Methods

### 4.1. Materials

Low-acyl Gellan Gum, GG (Lot: R13G030, ~500 kDa, Thermo Fisher Scientific, Ward Hill, MA, USA) was used as received. Styrene (St, ≥99%), 4-vinylbenzyl chloride (4-VBC, 90%), 2,2′-azobisisobutyronitrile (AIBN, 98%) were supplied by Millipore Sigma (St. Louis, MO, USA). St was used after activated alumina inhibitor removal, Al_2_O_3_ (activity grade I, ICN Biomedicals, Costa Mesa, CA, USA). Dimethyl sulfoxide (DMSO, EMD Millipore Corporation, Billerica, MA, USA), chloroform (J. T. Baker, Phillipsburg, NJ, USA), and methanol (Burdick and Jackson, Muskegon, MI, USA) were used as received. The following dyes (all from Aldrich, Milwaukee, WI, USA) were used in the binding experiments without further purification: Acridine Orange (AcOr, dye content ~90%), Auramine O (AO, ~85%), Bromphenol Blue (BPB, ~95%), Crystal Violet (CV, ~98%), Methylene Blue (MB, 98%), and Methyl Orange (MO, ~93%).

### 4.2. Methods

#### Modification of Gellan Gum

Firstly, 0.2 g of GG was dissolved in 3 mL anhydrous DMSO. A predetermined amount of 4-VBC, 1–10 equivalents (eq) per GG repeating unit, was then added, and the mixture was stirred for 10 min. To avoid side reactions with traces of moisture, several small portions of NaH on the tip of the spatula were added very last until gas (H_2_) evolution intensity was down (**Attention:** Protracted weighing of the solid NaH is not advisable—it reacts with the moisture in the air and could ignite). The mixture was stirred at room temperature for 24 h. After that, MeOH was added to quench the NaH excess and the solution was vacuum-filtered and washed with additional portions of MeOH and chloroform. The modified Gellan Gum (VBzGG-m) was obtained as a pale-yellow solid. Yields ranged from 96 to 98%.

### 4.3. Characterization of GG-m

#### 4.3.1. Fourier Transform Infrared (FT IR) Analysis

Bruker Tensor 27 IR instrument (Bruker Corporation, Billerica, MA, USA) was equipped with a MIR source and a DLaTGS detector. Spectra were recorded in the range of 4000–600 cm^−1^ under ambient conditions at a resolution of 4 cm^−1^. A total of 48 scans were recorded for each spectrum in addition to the background.

#### 4.3.2. Ultraviolet–Visible (UV–Vis) Spectroscopy

The degree of substitution (DS) was estimated using Agilent 8453 UV–Visible spectrophotometer (Agilent Technologies, Santa Clara, CA, USA). First, a calibration curve was constructed using a series of 4-VBC concentrations in DMSO (Figure S1). Then, the synthesized products were analyzed with pure DMSO as the blank solution.

#### 4.3.3. Nuclear Magnetic Resonance (NMR)

^1^H NMR spectra were recorded using D_2_O as a solvent at 22 °C with a Bruker AVANCE 600 MHz instrument (Bruker Co., Billerica, MA, USA). Tetramethylsilane was used as an internal standard. Number of scans—32.

### 4.4. Synthesis of SIPNs

Selected amounts of VBzGG-m, St, and AIBN were placed into a glass tube equipped with a magnetic stirrer. The amount of reagent was defined by the desired weight ratio of components (for example, VBzGG-1:St:AIBN = 1:100:0.5 weight% ratio was used for VBzGG-1 crosslinking). The reaction was conducted at 65 °C under a dry argon atmosphere for 6 h. After polymerization, the reaction mixture was cooled down to room temperature, extracted with CHCL_3_ for 3 days (the clear supernatant was removed every 24 h and the same volume of fresh CHCl_3_ was added), and the solvent swollen material was dried overnight in a vacuum oven at 50 °C. After the solvent evaporation, the amounts of the networks and extracted chloroform-soluble products were determined gravimetrically.

### 4.5. Characterization of SIPNs and Conetworks

#### 4.5.1. Swelling

The swelling behavior (the ability of the gel to retain certain liquids) was studied by a gravimetric procedure. A dry sample with known weight was immersed in chloroform or water at room temperature (RT). After certain periods of time, the swollen gel was weighted, and the swelling degree (SD) was calculated using the following Equation (1):

(1) $$\mathrm{SD}\;(\%)=[\left(W_{s}-W_{0})/W_{0}\right]\;\times \;100$$

where $W_{s}$ is the weight of the swollen gel at time t and $W_{0}$ is the weight of the dry gel.

#### 4.5.2. Size-Exclusion Chromatography (SEC)

The analyses of the extracted linear PSt were performed on a liquid chromatography line consisting of M510 pump, U6K universal injector, 486 tunable absorbance detector (Waters Corporation, Milford, MA, USA), and 250 dual refractometer/viscometer detector (Viscotek/Malvern Panalytical Ltd, Malvern, UK). The separation was carried out with a set of three 5 µm Styragel columns (HR 2, 3, and 5, Waters Corporation) and calibrated with 17 narrow dispersity PSt standards with molecular masses between 0.162 kDa and 956 kDa (Polymer Standards Service, Amherst, MA, USA).

#### 4.5.3. Thermal Gravimetric Analysis (TGA)

TGA was performed using a TA Q500 analyzer (TA Instruments, New Castle, DE, USA) in a temperature range of 25–600 °C at a heating rate of 10 °C/min and nitrogen flow 20 mL/min.

#### 4.5.4. Differential Scanning Calorimetry (DSC)

Differential scanning calorimeter DSC Q200, (TA Instruments, New Castle, DE, USA) was used to determine the glass transition temperatures (T_g_) and heat capacity jump (ΔC_p_) at the glass transition. Samples were heated in a heating–cooling–heating cycle from 0 to 180 °C at a scanning rate of 10 °C/min using dry nitrogen flow. The second heating cycle was used to determine the T_g_ of each sample.

#### 4.5.5. Dynamic Mechanical Analysis (DMA)

DMA was performed using a TA Q800 analyzer (TA Instruments, New Castle, DE, USA) with DMA Multi-strain module type and single cantilever clamp in a temperature range of 0–150 °C at a heating rate of 1 °C/min and a frequency of 5 Hz. Specimens with typical dimensions 25 (l)/12 (w)/2 (th) mm were prepared via thermal bulk radical copolymerization using silicone molds.

#### 4.5.6. Rheology Measurements

Rheological experiments were carried using Discovery Hybrid Rheometer (DHR3) with temperature-controlled lower Peltier plate geometry 8 mm (TA Instruments, New Castle, DE, USA). Swollen samples had a thickness of 2.0 mm. Frequency sweep experiments were performed on the hydrogels at 25 °C in the range 0.01–60 rad/s. Experiments were carried out in triplicate and the calculated values are reported as an average value.

#### 4.5.7. Scanning Electron Microscopy (SEM)

The surface morphology of swollen PSt-l-VBzGG-m conetworks was examined by a JSM 5800LV scanning electron microscope (JEOL, Tokyo, Japan). Samples were immersed and swollen in water or chloroform for 24 h, cryo-fractured, and carbon-coated under vacuum before the SEM micrographs were recorded.

#### 4.5.8. Dye Binding

Dry pieces of extracted 10 wt% PSt/GG-7 gel (5.68–12.28 mg) were immersed in dye solutions (1.31 ×10^−6^–1.63 × 10^−6^ mg/mL) and the UV spectra of the supernatant solutions were recorded after 24 h. The measured intensities were inserted in the Bouguer–Beer–Lambert equation [47], using previously established molar extinction coefficients [48].

## Figures and Tables

**Table 1:** Copolymerization conditions, amount of CHCl_3_ extracted substances and conetwork (gel) yields and swelling degree (SD) of conetworks in CHCl_3_ and water.

Macromonomer	VBzGG-m wt%	St wt%	AIBN wt%	Gel Yield %	Extractables ^a^ %	SD in CHCl_3_ %	SD in Water %
VBzGG-1	1	98.5	0.5	81.47	18.53	1037 ± 204	139 ± 98
	5	94.5	0.5	80.77	19.23	831 ± 190	338.125
	10	89.5	0.5	77.66	22.34	598 ± 98	487 ± 131
VBzGG-3	1	98.5	0.5	78.27	21.73	2279 ± 184	4955 ± 124
	5	94.5	0.5	77.96	22.04	2117 ± 330	529 ± 115
	10	89.5	0.5	81.35	18.65	681 ± 118	602 ± 97
VBzGG-5	1	98.5	0.5	80.97	19.03	2959 ± 254	552 ± 143
	5	94.5	0.5	78.44	21.56	2157 ± 105	601 ± 98
	10	89.5	0.5	75.44	24.56	1521 ± 186	659 ± 101
VBzGG-7	1	98.5	0.5	83.93	16.07	3556 ± 356	590 ± 102
	5	94.5	0.5	81.25	18.48	2216 ± 204	628 ± 124
	10	89.5	0.5	78.44	21.56	1639 ± 89	668 ± 137
VBzGG-10	1	98.5	0.5	76.00	24.00	3323 ± 419	603 ± 130
	5	94.5	0.5	77.65	22.35	2413 ± 388	633 ± 98
	10	89.5	0.5	80.16	19.84	1424 ± 150	795 ± 133

**^a^** Average total amount of substances extracted with CHCl_3_ after three days from three different specimens.

**Table 2:** Glass transition temperature (T_g_) and heat capacity jump (ΔC_p_). VBzGG-m amount: 1 wt%.

Macromonomer	Material	T_g_, °C	ΔC_p_, J/g °C
VBzGG-1	PSt extracted	92.4	1.71
	PSt-VBzGG-1 SIPN	87.2	1.03
	PSt-*l*-VBzGG-1	105.7	2.15
VBzGG-3	PSt extracted	91.3	0.60
	PSt-VBzGG-3 SIPN	95.7	0.59
	PSt-*l*-VBzGG-3	107.1	1.69
VBzGG-5	PSt extracted	88.7	0.49
	PSt-VBzGG-5 SIPN	97.0	0.90
	PSt-*l*-VBzGG-5	107.0	2.12
VBzGG-7	PSt extracted	91.5	1.04
	PSt-VBzGG-7 SIPN	64.7	1.24
	PSt-*l*-VBzGG-7	106.8	1.17
VBzGG-10	PSt extracted	89.8	0.35
	PSt-VBzGG-10 SIPN	96.0	0.69
	PSt-*l*-VBzGG-10	106.6	0.88
Linear PSt	synthesized at the same conditions	94.3	0.88
	formed in the presence of GG	93.4	0.35

## Data Availability

All results obtained in this study are included in the paper and in the Supporting Information. Any questions or requests can be referred to the corresponding author.

## Supplementary Materials

The following supporting information can be downloaded at: https://www.mdpi.com/article/10.3390/gels8100607/s1, Figure S1. Calibration curve constructed using UV–VIS absorbance of 4-VBC at 265 nm to estimate the Gellan Gum degree of substitution. Figure S2. Size-exclusion chromatography dRI traces of PSt extracted by CHCl_3_ after 48 h from PSt-VBzGG-m 1 wt% s-IPNs. A—VBzGG-1, B—VBzGG-3, C—VBzGG-5, D—VBzGG-7, E—VBzGG-10. Tol—toluene flow rate marker. Figure S3. FT-IR spectra of PSt-VBzGG-3 1 wt% SIPN (A) and PSt-*l*-VBzGG-3 1 wt% conetwork (B). Figure S4. Size-exclusion chromatography dRI traces of PSt isolated after 48 h of CHCl_3_ extraction of VBzGG-3 copolymerization mixtures. A—1 wt%; B—5 wt%, C—10 wt%. Tol—toluene flow rate marker. Figure S5. TGA thermograms of Gellan Gum (GG), extracted poly(styrene), PSt and PSt-VBzGG-m SIPNs (PSt-GG-m). Figure S6. DSC thermograms of PSt-VBzGG-5 1 wt% SIPN (A), PSt extracted from PSt-VBzGG-5 1 wt% SIPN (B) and PSt-*l*-VBzGG-5 1 wt% (C). Figure S7. Storage modulus of PSt-VBzGG-m SIPNs (GG-1–GG-10), PSt extracted from PSt-VBzGG-m SIPNs, and poly(styrene)/Gellan Gum physical mixture (PSt/GG). Figure S8. Loss modulus of PSt-VBzGG-m SIPNs (GG-1–GG-10), PSt extracted from PSt-VBzGG-m SIPNs, and poly(styrene)/Gellan Gum physical mixture (PSt/GG). Figure S9. Tan δ curves of PSt-GG-m SIPNs, PSt extracted from PSt-VBzGG-m SIPNs (GG-1–GG-10), and poly(styrene)/Gellan Gum physical mixture (PSt/GG). Figure S10. SEM of a PSt-*l*-VBzGG-5 10 wt% gel swollen in water. (a) sample at 200× magnification; (b) orange-framed area in image (a) observed at 850× magnification. Figure S11. Chemical structures of Auramine O (AO) and Bromphenol Blue BPB.
