# Supplementary material for: Stronger Together. Poly(Styrene) Gels Reinforced by Soft Gellan Gum
Source: Gels. 2022 Sep 22;8(10):607. doi: 10.3390/gels8100607 (PMC9601398; doi:10.3390/gels8100607)
Supplement: Supplementary file 1 [file gels-08-00607-s001.zip › gels-1917769-supplementary.pdf]

Supporting Information

Stronger Together. Poly(styrene) Gels Reinforced by Soft Gellan Gum

Dariya Getya[1,2] Ivan Gitsov[1,2,3]

[1] Department of Chemistry, State University of New York – ESF, Syracuse, NY 13210, USA
[2] The Michael M. Szwarc Polymer Research Institute, Syracuse, NY 13210, USA
[3] The BioInspired Institute, Syracuse University, Syracuse, NY 13244, USA

Table of contents:

Figure S1. Calibration curve constructed using UV-VIS absorbance of 4-VBC at 265 nm to estimate the Gellan Gum degree of substitution. Page 2

Figure S2. Size-exclusion chromatography dRI traces of PSt extracted by $CHCl_3$ after 48 h from PSt-VBzGG-m 1 wt% SIPNs. A – VBzGG-1, B – VBzGG-3, C – VBzGG-5, D – VBzGG-7, E – VBzGG-10. Tol – toluene flow rate marker. Page 2

Figure S3. FT-IR spectra of PSt-VBzGG-3 1 wt % SIPN (A) and PSt-*l*-VBzGG-3 1 wt% conetwork (B). Page 3

Figure S4. Size-exclusion chromatography dRI traces of PSt isolated after 48 h of $CHCl_3$ extraction of VBzGG-3 copolymerization mixtures. A - 1 wt%; B – 5 wt%, C – 10 wt%. Tol – toluene flow rate marker. Page 3

Figure S5. TGA thermograms of Gellan Gum (GG), extracted poly(styrene), PSt and PSt-VBzGG-m SIPNs (PSt-GG-m). Page 4

Figure S6. DSC thermograms of PSt-VBzGG-5 1 wt% SIPN (A), PSt extracted from PSt-VBzGG-5 1 wt% SIPN (B) and PSt-*l*-VBzGG-5 1 wt% (C). Page 4

Figure S7. Storage modulus of PSt-VBzGG-m SIPNs (GG-1 – GG-10), PSt extracted from PSt-VBzGG-m SIPNs, and poly(styrene)/Gellan Gum physical mixture (PSt/GG). Page 5

Figure S8. Loss modulus of PSt-VBzGG-m SIPNs (GG-1 – GG-10), PSt extracted from PSt-VBzGG-m SIPNs, and poly(styrene)/Gellan Gum physical mixture (PSt/GG). Page 5

Figure S9. Tan δ curves of PSt-GG-m SIPNs, PSt extracted from PSt-VBzGG-m SIPNs (GG-1 – GG-10), and poly(styrene)/Gellan Gum physical mixture (PSt/GG). Page 6

Figure S10. SEM of a PSt-*l*-VBzGG-5 10 wt % gel swollen in water. (a) sample at 200×magnification; (b) orange-framed area in image (a) observed at 850× magnification. Page 6

Figure S11. Chemical structures of Auramine O (AO) and Bromphenol Blue BPB Page 6

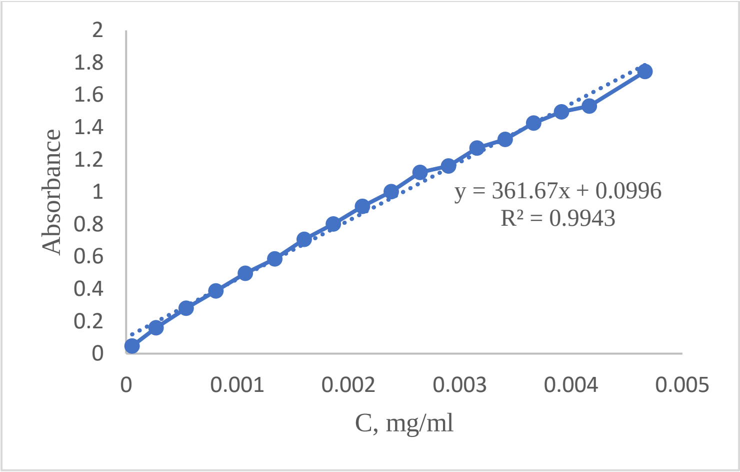

Figure S1. Calibration curve constructed using UV-VIS absorbance of 4-VBC at 265 nm to estimate the Gellan Gum degree of substitution.

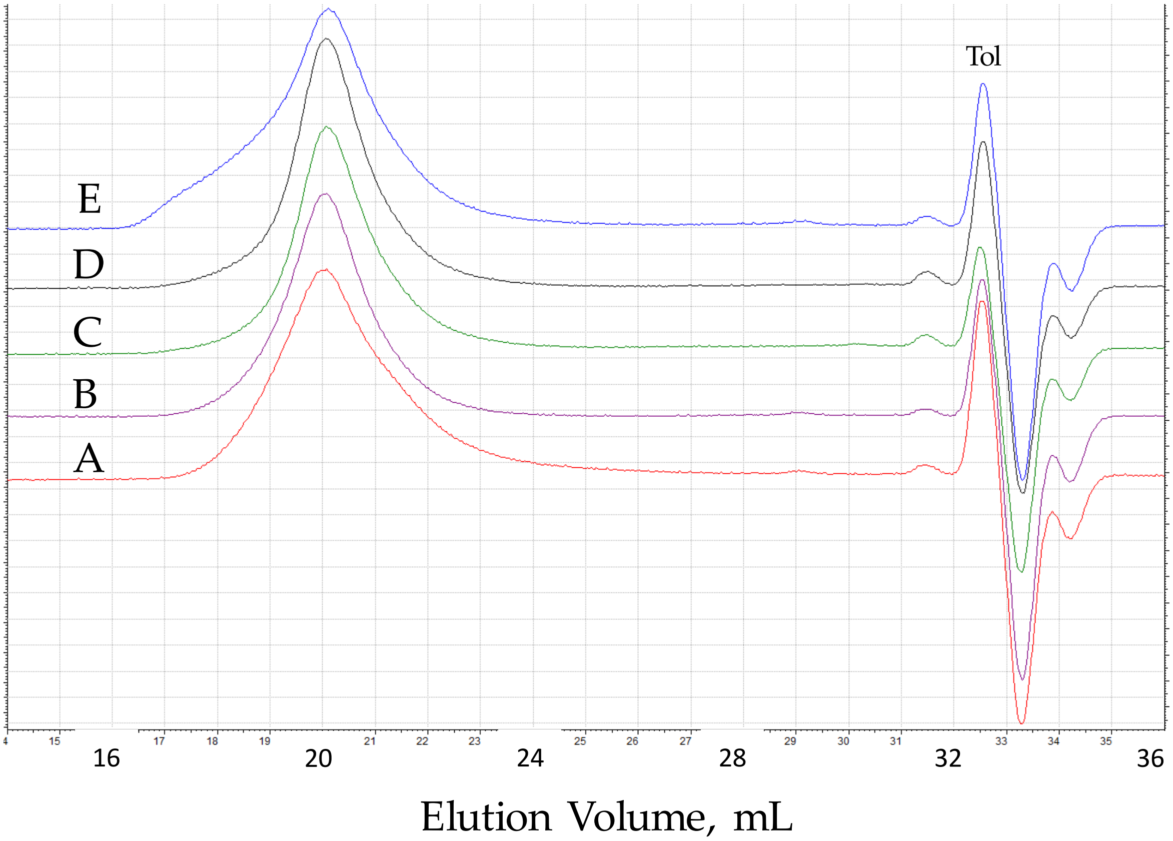

Figure S2. Size-exclusion chromatography dRI traces of PSt extracted by $CHCl_3$ after 48 h from PSt-VBzGG-m 1 wt% SIPNs. A – VBzGG-1, B – VBzGG-3, C – VBzGG-5, D – VBzGG-7, E – VBzGG-10. Tol – toluene flow rate marker.

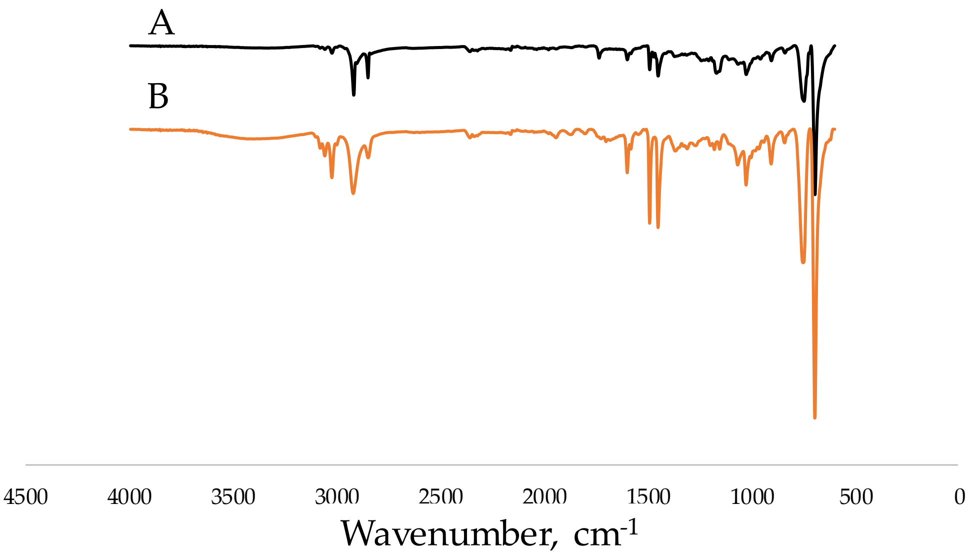

Figure S3. FT-IR spectra of PSt-VBzGG-3 1 wt % s- SIPN (A) and PSt-*l*-VBzGG-3 1 wt% conetwork (B).

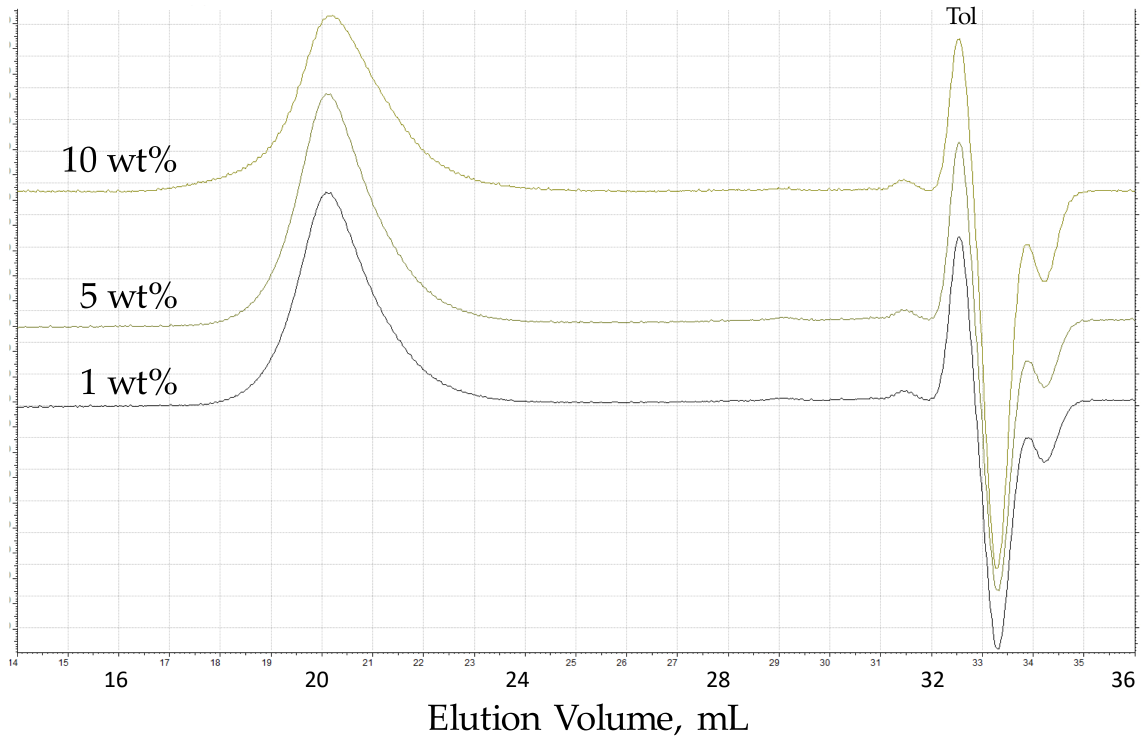

Figure S4. Size-exclusion chromatography dRI traces of PSt isolated after 48 h of $CHCl_3$ extraction of VBzGG-3 copolymerization mixtures. A - 1 wt%; B – 5 wt%, C – 10 wt%. Tol – toluene flow rate marker.

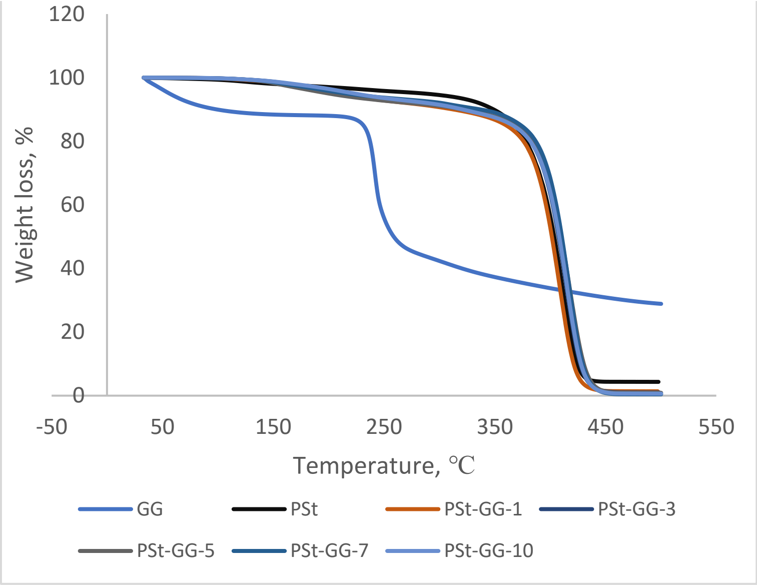

Figure S5. TGA thermograms of Gellan Gum (GG), extracted poly(styrene), PSt and PSt-VBzGG-m SIPNs (PSt-GG-m).

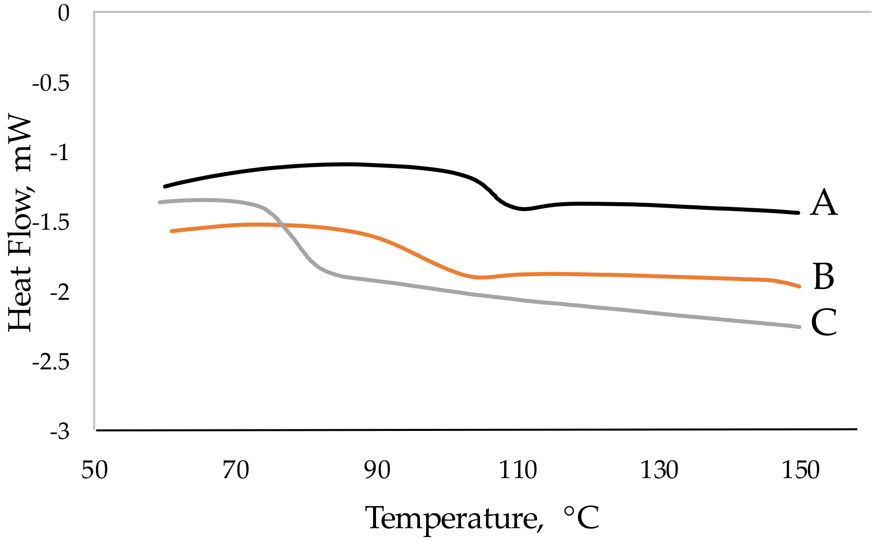

Figure S6. DSC thermograms of PSt-VBzGG-5 1 wt% SIPN (A), PSt extracted from PSt-VBzGG-5 1 wt% SIPN (B) and PSt-*l*-VBzGG-5 1 wt% (C).

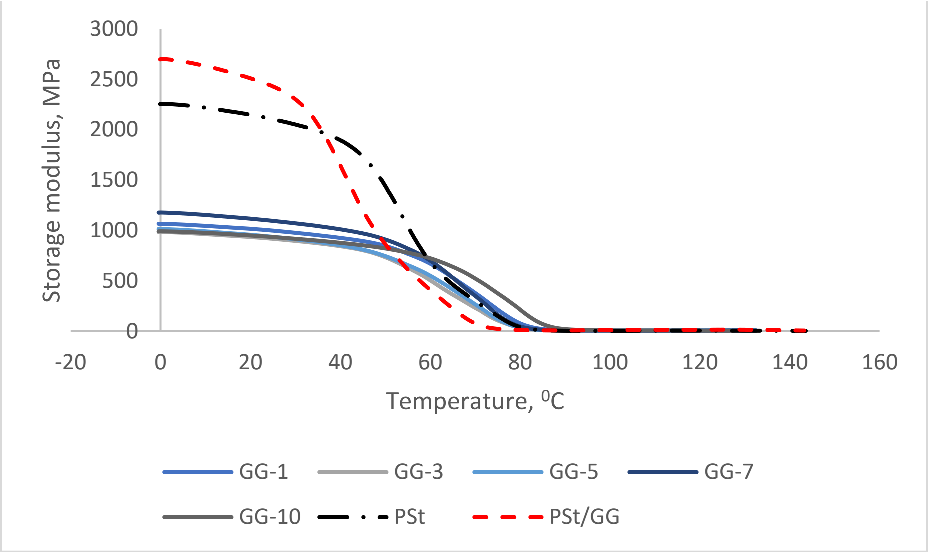

Figure S7. Storage modulus of PSt-VBzGG-m SIPNs (GG-1 – GG-10), PSt extracted from PSt-VBzGG-m SIPNs, and poly(styrene)/Gellan Gum physical mixture (PSt/GG).

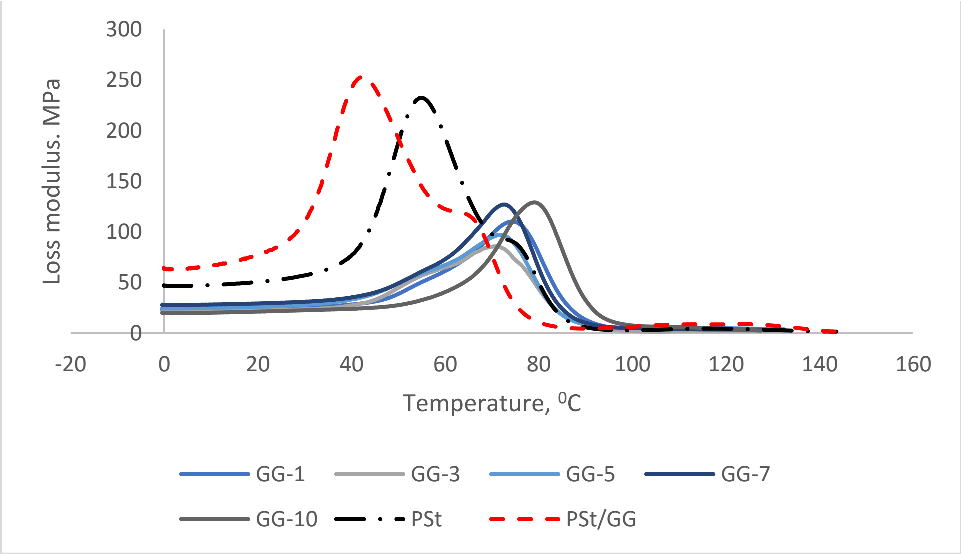

Figure S8. Loss modulus of PSt-VBzGG-m SIPNs (GG-1 – GG-10), PSt extracted from PSt-VBzGG-m SIPNs, and poly(styrene)/Gellan Gum physical mixture (PSt/GG).

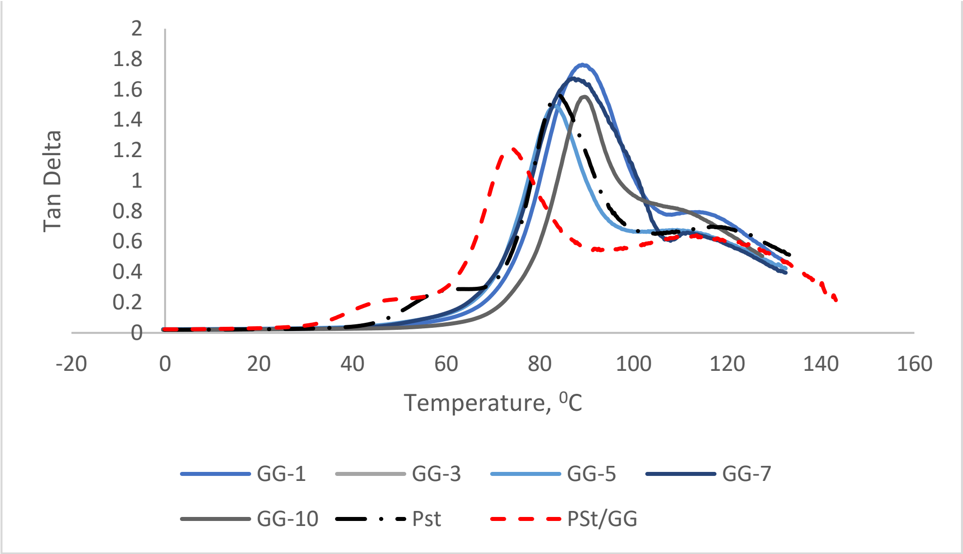

Figure S9. Tan δ curves of PSt-GG-m SIPNs, PSt extracted from PSt-VBzGG-m SIPNs (GG-1 – GG-10), and poly(styrene)/Gellan Gum physical mixture (PSt/GG).

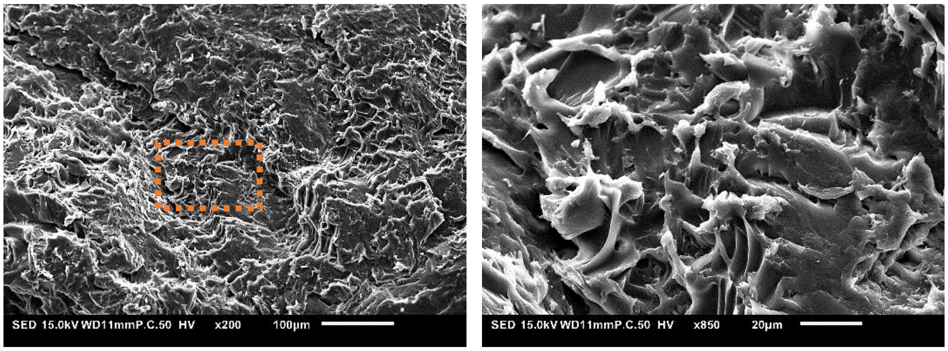

(a) (b)

Figure S10. SEM of a PSt-*l*-VBzGG-5 10 wt % gel swollen in water. (a) sample at 200× magnification; (b) orange-framed area in image (a) observed at 850× magnification.

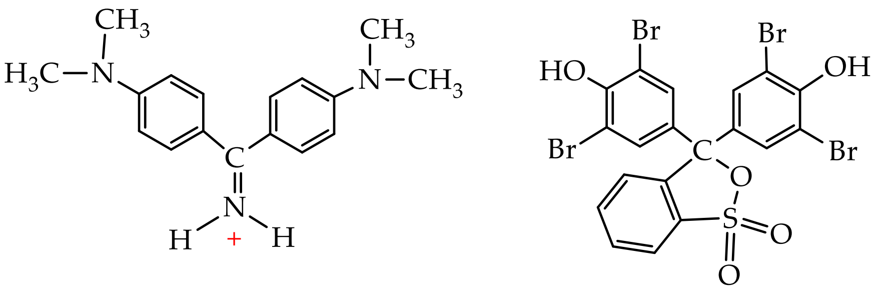

AO BPB

Figure S11. Chemical structures of Auramine O (AO) and bromphenol blue (BPB).
